# Supplementary material for: Experts’ Opinion for Improving Pertussis Vaccination Rates in Adolescents and Adults: A Call to Action
Source: Int J Environ Res Public Health. 2022 Apr 6;19(7):4412. doi: 10.3390/ijerph19074412 (PMC8998413; doi:10.3390/ijerph19074412)
Supplement: Supplementary file 1 [file ijerph-19-04412-s001.zip › ijerph-1603531-supplementary.pdf]

Table S1. Composition and brand of available dTap, DTPa-IPV and dTpa-IPV vaccines (number of pertussis antigens).

Ag	dTap (1) AJ Vaccines	dTap (3) GSK	dTpa (5) Sanofi	DTaP-IPV (3) GSK	dTap-IPV (3) GSK	DTaP-IPV (2) Sanofi	dTap-IPV (5) Sanofi
**Diptheria toxoid**	not less than 2 IU	not less than 2 IU	not less than 2 IU	not less than 30 IU	not less than 2 IU	not less than 30 IU	not less than 2 IU
**Tetanus toxoid**	not less than 20 IU	not less than 20 IU	not less than 20 IU	not less than 40 U IU	not less than 20 IU	not less than 40 IU	not less than 20 IU
**Pertussis toxoid (PT)**	20 g	8 g	2,5 g	25 g	8 g	25 g	2,5 g
**Filamentous haemagglutinin**	---	8 g	5 g	25 g	8 g	25 g	5 g
**Pertactin**	---	2,5 g	3 g	8 g	2,5 g	---	3 g
**Fimbriae types 2 and 3**	---	---	5 g	---	---	---	5 g
**Inactivated poliovirus type 1**	---	---	---	40 units	40 units	40 units	40 units
**Inactivated poliovirus type 2**	---	---	---	8 units	8 units	8 units	8 units
**Inactivated poliovirus type 3**	---	---	---	32 units	32 units	32 units	32 units

dTap = diphtheria-tetanus-acellular pertussis vaccine with reduced antigen content; DTaP-IPV = diphtheria-tetanus-acellular pertussis-inactivated poliovirus vaccine with full antigenic content; dTap-IPV = diphtheria-tetanus-acellular pertussis vaccine with reduced antigen content combined with inactivated poliovirus vaccine.
